# Supplementary material for: The role of fluctuations in determining cellular network thermodynamics
Source: PLoS One. 2020 Mar 11;15(3):e0230076. doi: 10.1371/journal.pone.0230076 (PMC7065797; doi:10.1371/journal.pone.0230076)
Supplement: Attachment — Submitted filename: PONE-D-19-26315_reviewer_commented.pdf [file pone.0230076.s002.pdf]

PLOS ONE

Properties of a Multidimensional Landscape Model for Determining Cellular Network Thermodynamics

--Manuscript Draft--

**Manuscript Number:**	PONE-D-19-26315
**Article Type:**	Research Article
**Full Title:**	Properties of a Multidimensional Landscape Model for Determining Cellular Network Thermodynamics
**Short Title:**	Cellular Network Thermodynamics
**Corresponding Author:**	Anne L Plant<br>National Institute of Standards and Technology<br>Gaithersburg, MD UNITED STATES
**Keywords:**	cellular network; statistical thermodynamics; potential landscape; cell population dynamics; Fokker-Planck; homeostasis; free energy; heat of dissipation; dynamic fluctuations
**Abstract:**	The steady state distributions of phenotypic responses within an isogenic population of cells result from both deterministic and stochastic characteristics of biochemical networks. A biochemical network can be characterized by a multidimensional potential landscape based on the distribution of responses and a diffusion matrix of the correlated dynamic fluctuations between N-numbers of intracellular network variables. The Boltzmann H-function defines the rate of free energy dissipation of a network system and provides a framework for determining the heat associated with the nonequilibrium steady state and its network components. We conjecture that there is an upper limit to the rate of dissipative heat produced by a biological system, and we show that the dissipative heat has a lower bound. The magnitudes of the landscape gradients and the dynamic correlated fluctuations of network variables are experimentally accessible, and through an analysis that we refer to as Thermo-Fokker-Planck (Thermo-FP), provide insight into the composition of the network and the relative thermodynamic contributions from network components. We surmise that these thermodynamic quantities allow determination of the relative importance of network components to overall network control.
**Order of Authors:**	Joseph B Hubbard
	Michael Halter
	Anne L Plant
**Additional Information:**	
**Question**	**Response**
**Financial Disclosure**<br><br>Enter a financial disclosure statement that describes the sources of funding for the work included in this submission. Review the submission guidelines for detailed requirements. View published research articles from *PLOS ONE* for specific examples.<br><br>This statement is required for submission and **will appear in the published article** if the submission is accepted. Please make sure it is accurate.	The authors received no specific funding for this work.

Powered by Editorial Manager® and ProduXion Manager® from Aries Systems Corporation

**Unfunded studies**<br>Enter: *The author(s) received no specific funding for this work.*<br><br>**Funded studies**<br>Enter a statement with the following details:<br>• Initials of the authors who received each award<br>• Grant numbers awarded to each author<br>• The full name of each funder<br>• URL of each funder website<br>• Did the sponsors or funders play any role in the study design, data collection and analysis, decision to publish, or preparation of the manuscript?<br>• **NO** - Include this sentence at the end of your statement: *The funders had no role in study design, data collection and analysis, decision to publish, or preparation of the manuscript.*<br>• **YES** - Specify the role(s) played.<br><br>* typeset	
**Competing Interests**<br><br>Use the instructions below to enter a competing interest statement for this submission. On behalf of all authors, disclose any competing interests that could be perceived to bias this work—acknowledging all financial support and any other relevant financial or non-financial competing interests.<br><br>This statement **will appear in the published article** if the submission is accepted. Please make sure it is accurate. View published research articles from *PLOS ONE* for specific examples.	The authors have declared that no competing interests exist.

**NO authors have competing interests**<br><br>Enter: *The authors have declared that no competing interests exist.*<br><br>**Authors with competing interests**<br><br>Enter competing interest details beginning with this statement:<br><br>*I have read the journal's policy and the authors of this manuscript have the following competing interests: [insert competing interests here]*<br><br>* typeset	
**Ethics Statement**<br><br>Enter an ethics statement for this submission. This statement is required if the study involved:<br><br>• Human participants<br>• Human specimens or tissue<br>• Vertebrate animals or cephalopods<br>• Vertebrate embryos or tissues<br>• Field research<br><br>Write "N/A" if the submission does not require an ethics statement.<br><br>General guidance is provided below. Consult the submission guidelines for detailed instructions. **Make sure that all information entered here is included in the Methods section of the manuscript.**	N/A

Powered by Editorial Manager® and ProduXion Manager® from Aries Systems Corporation

**Format for specific study types**<br><br>**Human Subject Research (involving human participants and/or tissue)**<br>• Give the name of the institutional review board or ethics committee that approved the study<br>• Include the approval number and/or a statement indicating approval of this research<br>• Indicate the form of consent obtained (written/oral) or the reason that consent was not obtained (e.g. the data were analyzed anonymously)<br><br>**Animal Research (involving vertebrate animals, embryos or tissues)**<br>• Provide the name of the Institutional Animal Care and Use Committee (IACUC) or other relevant ethics board that reviewed the study protocol, and indicate whether they approved this research or granted a formal waiver of ethical approval<br>• Include an approval number if one was obtained<br>• If the study involved *non-human primates*, add *additional details* about animal welfare and steps taken to ameliorate suffering<br>• If anesthesia, euthanasia, or any kind of animal sacrifice is part of the study, include briefly which substances and/or methods were applied<br><br>**Field Research**<br><br>Include the following details if this study involves the collection of plant, animal, or other materials from a natural setting:<br>• Field permit number<br>• Name of the institution or relevant body that granted permission	
**Data Availability**<br><br>Authors are required to make all data underlying the findings described fully available, without restriction, and from the time of publication. PLOS allows rare exceptions to address legal and ethical concerns. See the PLOS Data Policy and FAQ for detailed information.	No - some restrictions will apply

A Data Availability Statement describing where the data can be found is required at submission. Your answers to this question constitute the Data Availability Statement and **will be published in the article**, if accepted.<br><br>**Important:** Stating 'data available on request from the author' is not sufficient. If your data are only available upon request, select 'No' for the first question and explain your exceptional situation in the text box.<br><br>Do the authors confirm that all data underlying the findings described in their manuscript are fully available without restriction?	
**Describe where the data may be found in full sentences. If you are copying our sample text, replace any instances of XXX with the appropriate details.**<br><br>• If the data are **held or will be held in a public repository**, include URLs, accession numbers or DOIs. If this information will only be available after acceptance, indicate this by ticking the box below. For example: *All XXX files are available from the XXX database (accession number(s) XXX, XXX.).*<br>• If the data are all contained **within the manuscript and/or Supporting Information files**, enter the following: *All relevant data are within the manuscript and its Supporting Information files.*<br>• If neither of these applies but you are able to provide **details of access elsewhere**, with or without limitations, please do so. For example:<br><br>*Data cannot be shared publicly because of [XXX]. Data are available from the XXX Institutional Data Access / Ethics Committee (contact via XXX) for researchers who meet the criteria for access to confidential data.*<br><br>*The data underlying the results presented in the study are available from (include the name of the third party*	No data are used in this theoretical study.

and contact information or URL).<br>• This text is appropriate if the data are owned by a third party and authors do not have permission to share the data.<br><br>* typeset	
Additional data availability information:	

Powered by Editorial Manager® and ProduXion Manager® from Aries Systems Corporation

manuscript Click here to download Manuscript for PLOSText.docx

Properties of a Multidimensional Landscape Model for Determining Cellular Network Thermodynamics

Joseph B. Hubbard[1], Michael Halter[1], Anne L. Plant[1*]

[1] Biosystems and Biomaterials Division, National Institute of Standards and Technology, Gaithersburg MD, United States of America

[*]Corresponding author

E-mail: anne.plant@nist.gov

ABSTRACT

The steady state distributions of phenotypic responses within an isogenic population of cells result from both deterministic and stochastic characteristics of biochemical networks. A biochemical network can be characterized by a multidimensional potential landscape based on the distribution of responses and a diffusion matrix of the correlated dynamic fluctuations between N-numbers of intracellular network variables. The Boltzmann H-function defines the rate of free energy dissipation of a network system and provides a framework for determining the heat associated with the nonequilibrium steady state and its network components. We conjecture that there is an upper limit to the rate of dissipative heat produced by a biological system, and we show that the dissipative heat has a lower bound. The magnitudes of the landscape gradients and the dynamic correlated fluctuations of network variables are experimentally accessible, and through an analysis that we refer to as Thermo-Fokker-Planck (Thermo-FP), provide insight into the composition of the network and the relative thermodynamic contributions from network components. We surmise that these thermodynamic quantities allow determination of the relative importance of network components to overall network control.

if this is the main result it might be used for the title

INTRODUCTION

Measurements of individual cells within a population indicate that phenotypic differences between

refs

isogenic cells is common, even when they are in a homogeneous and stable environment. Despite differences between individuals, populations of cells can manifest apparently stable distributions of

refs?

phenotypic expression. The measured phenotypic parameters can include concentration of protein products of gene expression, indicators associated with promoter activation, RNA transcripts, and

behavior is more like signaling … morphology is a trait

complex cell behavior such as morphology. The steady state distribution of phenotypes observed from single cell analysis is a probability density function and can be represented as a potential energy

can be still debated …

landscape (1-5).

Studies that involve imaging of live cells reveal that despite the observed consistency of the steady state, or stationary, distribution of phenotypes across the population of cells, there can significant dynamic variability in each cell and from one cell to another (3, 6-11). A number of studies provide direct evidence that the distribution of phenotypes in steady state distributions is ergodic in that subsequent to a transient perturbation the population will eventually relax to the steady state distribution (3, 12-16). The recapitulation of the steady state distribution, even after single cell cloning demonstrates that each cell or its progeny can explore all microstates on the landscape. The population response appears deterministic, but the individual entities (cells) that comprise the population present a dynamic, random expression of phenotypes, which ultimately results in the heterogeneity of the population. These are characteristics of many physical systems that can be well-described by statistical mechanics. The variations in populations of cells is often attributed to stochastic fluctuations, or noise, due to small numbers of molecules associated with transcription and translation. However, fluctuations in small numbers of molecules is not the only source of this variation (8, 17-19); correlation analysis has suggested that the main source of noise may be upstream regulatory components (12). Ensembles of biochemicals are responsible for producing an observed phenotype (8, 19, 20), and these network components demonstrate coordinated concentration response functions. Importantly, dynamic fluctuations of network components, and correlations in fluctuations among multiple network components, are defining features of regulated networks (2, 14, 19, 21-27). An abbreviated biochemical network system is depicted in the schematic in Fig 1, based on the interactions between transcription factors involved in maintaining pluripotency (28), and provides an illustration of some of the general network features the our model addresses.

Fig 1. Schematic of an intracellular network This is based on a schematic by Rizzino and Wuebben 2016 (28) on control of pluripotency, and is consisting of N=3 variable components, ~~or dimensions,~~ of a network with activation and repression relationships as indicated. As described in the text, these relationships define the measurable covariances in an NxN diffusion matrix, which contribute to the thermodynamics of the network together with the gradients of a landscape (which are derived from the multi-dimensional steady state probability density). The nonequilibrium steady state in this open thermodynamic system is supported by an influx of free energy from outside the system, which is dissipated as heat.

Identification of network components and the nature of their interdependence remains a challenge. While "omics" analysis methods can probe many variables simultaneously and even at the level of not yet for all omics individual cells, they provide only a snapshot in time. While these methods allow determination of the coincidence of molecular species, without dynamic data in individual cells that can reveal the rates and magnitudes of the interactions between network species, it is impossible to determine unambiguously the relationship between them or to determine a physical mechanistic basis for control of the network (10, 19, 29, 30). A promising approach for observing single cell dynamics is imaging of live cells over time, which provides access to fluctuations in phenotypic expression in individual cells across a population (9-11, 14, 24). Live cell imaging can also provide spatial and temporal information across scales and can provide ancillary information such as direct observation of the timing of cell division. We have previously integrated used quantitative microscopy (3, 7) to track temporal responses of populations of individual cells, and Langevin/Fokker-Planck (L/FP) dynamics to analyze the population distributions as potential energy landscapes (3). By measuring real time trajectories of the activity of the gene promoter for the extracellular matrix protein tenascin C, we determined the diffusion coefficient for fluctuations in the promoter, and employing L/FP dynamics, accurately predicted the time dependent relaxation of subpopulations to the steady state distribution with no adjustable parameters (3). Such a one-dimensional landscape is a simple manifestation of a complex adaptive system but provides little insight into the mechanisms responsible for establishing and maintaining the stability and dynamics of the steady state distribution because of the contributions from unseen variables. In the current theoretical

work, we consider a network of N numbers of variables. An N-dimensional statistical thermodynamics analysis allows fundamental questions about the thermodynamic controls in a coordinated network system to be addressed, including: how to identify the most important components of a regulated network, what is the relative thermodynamic contribution of different network components, and what is the thermodynamic price of homeostasis of a regulated network?

We present an experimentally accessible theoretical Fokker-Planck (FP) framework, which we refer to as Thermo-FP, that demonstrates that the rate of free energy dissipation associated with maintaining a nonequilibrium steady state network of intracellular reactions can be determined by the covariances in the temporal fluctuations in the components of the network and the gradients of the potential landscape. This analysis is enabled a by recent application of FP dynamics (31) to demonstrate that the Boltzmann H-function explicitly connects the relative entropy of a relaxing population with the rate of dissipation of free energy involved in maintaining the network at steady state.

RESULTS

The theoretical framework

not necessarily to transient perturbation - also steady state solutions or general dynamics. Approximation of Master Eq.

The Fokker-Planck (FP) equation (32) describes the time evolution of the probability density of a population as it approaches a steady state distribution following a transient perturbation. This evolution is considered at small time intervals as a function of a deterministic component in the form of a drift vector, and a diffusion component that describes the stochastic fluctuations within the distribution. We

why thermo? The diffusion term represents in general noise such as thermal noise (temperature)?

present Thermo-FP dynamics to describe the evolution to a steady state of an N-dimensional probability distribution with N x N diffusion matrices of variances and covariances in and between network

variables. The magnitude of the coupled fluctuations between the different network variables, i.e., the covariances that comprise the diffusion matrix, is a measure of the strength of the physical and functional interaction between those variables (19). The steady state is a relatively low free energy, high entropy state. The fluctuations that occur around this state are states of higher free energy and lower entropy, and the magnitude of the measurable correlated fluctuations between network variables, which comprise the diffusion matrix, is related to heat through the Boltzmann constant (33). The coordinated relationships between network components create an organized structure, thus reducing entropy and resulting in the production of dissipative heat (34).

how can fluctuations represent a state here? what does higher/lower mean here? relative to what?

Here we treat multidimensional landscapes of biochemical networks by applying the mathematical properties of positive definite quadratic forms and normal mode analysis common to mathematical physics and statistical mechanics (35). A diffusion matrix of the dynamic covariances between the multiple variables of the network can be rotated through normal mode analysis to identify complex collective modes of network variables. The product of an eigenvalues of the rotated diffusion matrix with the square of the gradient of the rotated multidimensional landscape allows determination of the contribution each degree of freedom makes to the rate of dissipation of heat that maintains the regulated network of reactants.

needs better explanation! How to treat Wentzell-Freidlin potentials with non-vanishing gradient component?

We begin by showing that the Boltzmann H function describes the free energy of a population relaxing by Thermo-FP dynamics to a steady state distribution.

The steady state landscape and the Boltzmann H Function

The Boltzmann equation relates entropy, S, to the number of microstates in a distribution, W, of an equilibrated system: $S = k_B \ln W$. For a nonequilibrium steady state system, the occupation of

this doesn't hold true - see concepts from stochastic thermodynamics (Jarzynski, Evens, Seifert, Esposito - partly references in SI)

microstates results from irreversible processes and the steady state is supported by influx of external free energy. For a multi-dimensional system of N network variables that is approaching its nonequilibrium steady state, entropy decreases over time: $S = -\int W(\{x\},t) \ln W(\{x\},t)\, d^N x$ where $W(\{x\},t)$ is the occupation probability density of phenotypic microstates as a function of time and location on the N-dimensional landscape. For both equilibrium and nonequilibrium systems, a minimum free energy is achieved when a system has relaxed to its stable steady state. During relaxation, which is

definition Eq. 1 should be here!

dependent on stochastic fluctuations, relative free energy is dissipated as heat. The free energy of a system approaching equilibrium can be identified with the Boltzmann H-function, $H(t)$, and $-{dH}/{dt}$ is the rate of dissipation of free energy as a system that is transiently perturbed away from its steady state relaxes to a steady state distribution, W_{ss}. In the following presentation, the factor kT is assumed but not explicitly included in equations.

this should be set in the context of the stoch. thermodynamic literature - specifically with the work of Rao et al who give a solid derivation for this: https://journals.aps.org/prx/abstract/10.1103/PhysRevX.6.041064

$H(t)$ is a relative free energy defined by

$$H(t,\tau) = \int d^N x\, W_1(\{x\},t) \ln \frac{W_1(\{x\},t)}{W_2(\{x\},t+\tau)} \qquad (1)$$

in terms of probability densities, where W_1 is the probability density of the microstates at some time, t during relaxation and W_2 is the probability density at time $t+\tau$ (depicted schematically in Fig. 2). Thus, the relative free energy of the system is defined by the average of the logarithm of the ratio of the occupation probabilities of the microstates, $\{x\}$, of the distribution. Keeping in mind that time-dependent relaxation is determined by stochastic fluctuations of characteristics rates, a series of probability density functions is observed over time (Fig. 1), each of which can be described by Thermo-

FP dynamics in terms of a potential force, or drift, term and a diffusive term at every microstate; the former corresponds to the gradient of the landscape on which the microstate resides, and the latter is described in an N by N diffusion matrix of variances and covariances of the fluctuations of the N network variables (see SI1). As the perturbed system relaxes, $H(t)$ decreases with time ($\frac{dH(t)}{dt} \leq 0$) and is a minimum at steady state, SS.

The time derivative of the Boltzmann H-function, $\frac{dH(t)}{dt}$ can be written as a quadratic form (31, 32), consistent with FP dynamics.

$$\frac{dH(t)}{dt} = -\frac{1}{2}\sum_{i,j}^{N} \int d^N x \, W(\{x\}, t) \frac{\partial}{\partial x_i} \ln R \cdot D_{ij}(\{x\}) \cdot \frac{\partial}{\partial x_j} \ln R \tag{2}$$

Here a measurable multi-dimensional probability density provides the relative free energy associated with the microstates of the landscape, $R = \frac{W(\{x\},t)}{W_{SS}(\{x\})}$. The gradients of the landscape, $\frac{\partial}{\partial x_i} \ln R$ and $\frac{\partial}{\partial x_j} \ln R$ are analogous to the gradients of a chemical potential; and D_{ij} is a diffusion matrix of experimentally determined covariances between the N variables (36, 37). The N-dimensional diffusion matrix, D_{ij}, reflects the fluctuations in each of N network components, which are the diagonal elements (variances), and the dynamic correlations (covariances) between the N variables, which are the off-diagonal elements.

Fig. 2. A steady state distribution, if transiently perturbed, will relax back to the steady state distribution. (Left) The rate of dissipation of free energy during relaxation, $\frac{dH(t)}{dt}$, approaches 0 as the steady state (W_{ss}) is reached. A thermodynamically open, nonequilibrium system is less entropic, and of higher free energy, than an equilibrium system. (Right) $H(t)$ corresponds to population distributions (shown here for 1-dimensional distributions) (from

Sisan et. al. 2012)(3). A subpopulation resulting from cell sorting (Day 0) is allowed to relax, and over time, the population achieves the initial steady state distribution from which the subpopulation was taken.

The energetic components of the non-equilibrium steady state

By setting $R(\{x\},t) = \frac{W(\{x\},t)}{W_{SS}(\{x\})}$ in Eq. 2, where $W_{SS}(\{x\})$ refers to a time-invariant steady state, we see that Thermo-FP dynamics ensures that $\frac{dH(t)}{dt}$ vanishes as the stationary state is approached and R approaches unity and $\ln R\,(\{x\},t)$ approaches 0. isn't this trivial?

At equilibrium, the rate of increase in entropy and the rate of dissipation of free energy will approach zero. Living biological systems do not exist at equilibrium, and instead reach a nonequilibrium steady state with reduced entropy and higher free energy compared to an equilibrium state. The equilibrium state is defined by detailed balance, in that the probability flux between any pair of points on the landscape is equal to the probability flux in the reverse direction; in the nonequilibrium state there is a lack of reversibility. Interactions between network components create a coordinated set of reactions; this allows the system to respond dynamically to arbitrary perturbations in order to recover homeostasis. This molecular organization reduces entropy and keeps the system in a nonequilibrium steady state that is maintained by the injection of free energy from outside the system; that energy is dissipated by the system as heat (34). During relaxation to an equilibrium steady state, $\frac{dH(t)}{dt}$ is the rate of free energy dissipated from the production of entropy during approach to the steady state, i.e., ${}^{\mathrm{dH}}/_{dt} = -\dot{Q}_{ENTROPY}$. During relaxation to a nonequilibrium steady state distribution, $\frac{dH(t)}{dt}$ is the rate of free energy dissipated from two sources, namely $\dot{Q}_{ENTROPY}$, and also the rate of dissipation of heat from the free energy that is required to maintain the nonequilibrium steady state, $\dot{Q}_{ENERGY}$. (The dot indicates the time rate of change of the quantity). Thus, for a nonequilibrum system:

$$\dot{Q}_{ENERGY}(t) \geq 0,\ \dot{Q}_{ENTROPY}(t) \geq 0$$

(3)

$$\frac{dH(t)}{dt} = \dot{Q}_{ENERGY}(t) - \dot{Q}_{ENTROPY}(t) \leq 0$$

why does this holds in general?

(4)

Before reaching steady state:

$$\frac{dH(t)}{dt} = \frac{1}{2}\sum_{i,j}^{N} \int d^N x\, W(\{x\},t) \left[\frac{\partial}{\partial x_i} \ln W_{ss}(\{x\}) \cdot D_{ij}(\{x\}) \cdot \frac{\partial}{\partial x_j} \ln W_{ss}(\{x\})\right] - \dot{Q}_{ENTROPY}(t) \leq 0$$

(5)

At steady state:

$$\dot{Q}_{ENERGY} = \frac{1}{2}\sum_{i,j}^{N} \int d^N x\, W(\{x\}) \left[\frac{\partial}{\partial x_i} \ln W_{ss}(\{x\}) \cdot D_{ij}(\{x\}) \cdot \frac{\partial}{\partial x_j} \ln W_{ss}(\{x\})\right] \geq 0$$

(6)

The Boltzmann H function thus provides us with a measure of the rate of heat dissipation, which we will call $\dot{Q}_{ENERGY}$, associated with maintaining the nonequilibrium steady state that is analogous to the "housekeeping heat" introduced by Oono and Paniconi (38) and further developed by others (39, 40). We define $\dot{Q}_{ENERGY}$ as distinct from housekeeping heat because it is the dissipative heat that remains associated with a nonequilibrium steady state after $\frac{dH(t)}{dt}$ has reached zero. It is a value that can be determined in the absence of consideration of an equilibrium distribution, which is not experimentally observable in a biological system. At steady state the rate of heat dissipation due to $\dot{Q}_{ENERGY}$ is equal to the rate of entropy production (39, 41).

Normal mode representation of the diffusion matrix

The nonequilibrium steady state distribution of a cell population can be thought of as composed of microstates, $\{x\}$, the different ways the population can achieve its continuum of phenotypic expressions. The phenotypic expressions of the population are determined by the dynamic interactions of the components of the network; these interactions are represented in the N x N diffusion matrix, $D_{ij}(\{x\})$, as the autocorrelations and the dynamic covariances of the fluctuations of the activities of the N network variables. The dynamic covariances are the mean square fluctuations about the average relative expression levels divided by time. The H function provides us with the time-derivative of the relative free energy of the system, from which we can identify $\dot{Q}_{ENERGY}(t)$, the rate at which heat is dissipated during the maintenance of the nonequilibrium steady state of the network. We will use these relationships to determine the relative rate of heat dissipation by the various network components to support the homeostatic steady state distribution.

The normal modes of the diffusion matrix result from rotation of the matrix ($D_{ij}(\{x\})$)to a diagonal form and provide us with a matrix ($D_{ii}(\{x^*\})$) in which the eigenvalues of all diagonal elements are greater than 0 and the off-diagonal elements are equal to 0, i.e., the components of the matrix are independent of one another. The purpose of this transformation is to identify combinations of network components as composite variables that are the major contributors to the rate of heat dissipation of the system; these are effectively the degrees of freedom. The original diffusion matrix represents the pair-wise interactions between network variables, some of which are positive and some negative. Rotation of the matrix allows us to define composite variables (degrees of freedom) as clusters of N-wise interactions

between the N components of the network. The rotation operation guarantees eigenvalues that are positive as well as eigenvectors that are mutually orthogonal. We transpose integration and summation (42) operations in Eq. 6 to yield an expression for the energetics of the system as

$$\dot{Q}_{ENERGY}(t) = \int d^N x^* \, W(\{x^*, t\}) \cdot \sum_{i}^{N} \lambda_i(\{x^*\}) \, V_i^2(\{x^*\}) \quad . \tag{7}$$

In Eq.7, we isolate terms within the summation operator to better illustrate their energetic contributions to the network. In Eq. 7, $\lambda_i(\{x^*\})$ are the eigenvalues of the rotated diffusion matrix and represent interactions between network components and the magnitude of their coordinated fluctuations. Eigenvectors represents ways, i.e., modes, in which network components are organized with respect to their interactions with one another. The eigenvalues are the diffusion coefficients associated with those independent collective modes of the network components. The term $V_i(\{x^*\})$ is equal to the gradient of the potential defined by the rotated landscape and expressed as $\left[V_i{}^2(\{x^*\})\right] = \left[\frac{\partial}{\partial x_i{}^*} \ln W_{ss}(\{x^*\})\right]^2$. It should be noted that the eigenvalues as well as the gradients of the potentials are functions of the entire coordinate set of x*. Eq. 7 shows that the rate of free energy dissipation to maintain the nonequilibrium steady state will be largest when eigenvalues are large because of strong dynamic interactions between components of the network and when those interactions are occurring in a part of the landscape that is characterized by steep gradients. The rotated diffusion matrix produces positive eigenvalues and corresponding orthogonal eigenvectors, which simplifies the next step which is to sum the most important contributors to the rate of free energy utilization and heat dissipation.

Network contributions to $\dot{Q}_{ENERGY}$

$\dot{Q}_{ENERGY}$ in Eq. 7 is the rate at which the N-dimensional network is dissipating heat associated with the maintenance of the nonequilibrium steady state. It can also be regarded as the rate at which external free energy is injected into the network. The summation term on the right side of Eq. 7 is a positive definite quadratic form; in keeping with statistical thermodynamics we use this term to determine the energy that each of the degrees of freedom contribute to the system. The magnitude of $\lambda_i(\{x^*\})$ and $[V_i(\{x^*\})]^2$ in $\dot{Q}_{ENERGY}$ integrated over the probability distribution defines the rate of heat dissipation by the various components of the homeostatic network. This treatment implies that some interactions between network components or variables are more dissipative than others and therefore are more important thermodynamic contributors to the steady state. Below we will address the significance of this.

If we consider each composite network variable that is identified by rotation of the matrix as a contributor of a degree of freedom, their sum, C, represents the total rate of heat dissipation associated with the system as shown in Eq. 8.

$$C = \sum_{i}^{N} \lambda_i [V_i^*]^2 \tag{8}$$

Each term is an implicit function of x*and reflects a heat that is dependent on the magnitudes of the eigenvalues of the matrix, λ_i, and the square of the gradient, V_i^*, of the landscape that corresponds to the rotated coordinate system. Together these terms constitute a quadratic representation of the microscopic dissipation of the regulated circuit, a sequence of partial sums that increases monotonically

as the number of terms increases. We are proposing that reactions that involve high rates of free energy dissipation are more important to the function of the cell vis a vis stability and adaptability.

We now make an assumption that for a biological system, there will likely be an upper bound, C_{UB} on the local rate of dissipative heat produced. This upper limit may arise from the temperature associated with heat generation or the limited rate of transport of energy or matter from the environment into the system. The value of the sum of the energetics of the network components thus cannot exceed the upper bound denoted by:

$$C_{UB} \geq C = \lambda_1 {V_1^*}^2 + \lambda_2 {V_2^*}^2 + \lambda_3 {V_3^*}^2 + \cdots \lambda_N {V_N^*}^2 \qquad (9)$$

Each of the elements in Eq. 9 is a contributor to the summation of network dissipative heat. This treatment thus provides us, in principle, with an experimentally tractable way of assessing the relative contribution that each multivariable component makes to the rate of heat dissipation in the maintenance of the network, and the free energy cost associated with keeping the network in homeostasis. When the number of network dimensions, N, is sufficiently large, C reaches a limit, C_{UB} and after integrating over the multidimensional probability distribution this constant rate is identical to $\dot{Q}_{ENERGY}$. A geometric approach for estimating the size of N required for convergence of C to C_{UB} is described in SI3.

In addition to the assumption of an upper bound on dissipative heat, we utilize a generalization of the maximum entropy principle to show that the homeostatic heat generation rate, $\dot{Q}_{ENERGY}$, also has a

lower bound, i.e., there exists a finite dissipative gap between the non-equilibrium stationary state and the equilibrium (detailed balance) distribution (see SI4).

DISCUSSION

While it has been frequently noted that stochastic fluctuations in molecular components in individual cells are important to regulatory mechanisms in one-dimensional systems (12, 24, 43, 44) and in multidimensional networks (21, 25, 27, 45, 46), to our knowledge, this work is the first to show the relationship between fluctuations in network variables and the thermodynamic quantities associated with the interactions between network components. We have limited our theoretical Thermo-FP approach to one that is experimentally tractable. We have shown here how the use of a potential landscape and a diffusion matrix provides a framework for determining the relative energetic contributions of the components of a regulated network. We derive an experimentally accessible value for $\dot{Q}_{ENERGY}$, the rate of heat dissipation associated with maintaining the nonequilibrium steady state and the analog of "housekeeping heat", directly from the Boltzmann H function. While we have presented this method as applied to analyzing steady state distributions, this approach is also applicable to population state transitions as discussed in SI1.

this should be set in the context of the literature such as e.g. Mojtahedi et. al https://journals.plos.org/plosbiology/article?id=10.1371/journal.pbio.2000640 and work from Chen et al https://www.nature.com/articles/srep00342

Large magnitudes of correlated fluctuations of network components are associated with large rates of heat dissipation (41), and our theoretical treatment shows that this is especially true when these fluctuations occur in areas of steep landscape gradients. Noise and complexity are defining features of biological systems, and the Thermo-FP analysis suggests a thermodynamic basis for the relationship between noise and complexity and the stability of a regulated network. Fluctuations are required to ensure ergodicity by allowing cells to escape from deep attractor basins and maintain the stability of the

entire landscape structure. We may consider that a biological system requires both stability and adaptability even though these may seem to be opposing characteristics. The Thermo-FP treatment presented here provides a thermodynamic basis for understanding why both the diffusion matrix and barrier gradients are important for maintaining the distribution of phenotypes. Deeper attractor basins associated with non-negligible diffusion coefficients enable stability of the network landscape during nominally constant environmental conditions, and shallower attractors allow adaptation to changing conditions through transition to a new steady state. Very small diffusion coefficients could result in long-lived metastable states, analogous to glassy conditions.

The reasonable assumption that there will be a limit upper to the rate of heat dissipation in a biological system suggests that characteristics of landscapes and diffusion coefficients provide insight into numbers of network variables, stability, and composition of the network. There exists a geometric estimation of the number, N, of variables required for convergence in terms of the volumes of N-balls and N-ellipsoids (47). Depending on characteristics of the system, such as roughness of the landscape, sufficient numbers of network terms can be predicted to be as small as a few, or many times larger. A reasonable estimate for the number of variables is between 8 and 10. How a sufficient number of terms for convergence to N can be determined is discussed in SI3. For example, very large landscape gradients that correspond to large eignenvalues would contribute strongly to the overall dissipative heat of the network, and a small number of such contributors may be sufficient to reach a thermal limit. This condition would be indicated by a landscape containing one or a small number of very deep attractor basins.

The measurement of dynamic correlations of many cellular variables over time in individual cells is in principle achievable with time-resolved fluorescence microscopy of live cells (see SI2 for details), especially when enabled by automation and advances in handling of large image datasets (48, 49).

Although transcriptomics analysis can probe a larger number of genes compared to live cell imaging, the appropriate interpretation of the relative significance of these changes to network function and their relationships to one another can be ambiguous (19, 29). Methods like transcriptomics analysis that rely on "snapshots" of populations at single points in time can infer temporal and treatment-dependent relationships between variables, but real time trajectories of changes in gene expression in individual cells, such as is accessible by live cell imaging, can provide unambiguous determination of correlations in

why do the authors then talk about single cell RNAseq in SI (the common abbreviation sc-RNAseq should be used there!)

stochastic fluctuations between network variables. In the absence of such direct measurements, a number of approaches have been pursued for inferring individual cell trajectories from population data including population balance analysis (29), optimum transport (50), and others discussed in (50). For these methods to be analytically tractable, a simplifying assumption is made that the stochastic component is Gaussian and uniform over gene expression space (as discussed further in SI1).

In Thermo-FP, stochasticity is captured in in the dynamic fluctuations of the network variables. The magnitudes of the correlations in dynamic fluctuations provide a direct measure of the thermodynamics of network component interactions (since the magnitudes of the fluctuations are proportional to $k_B T$, Boltzmann's constant times temperature), and thus the relative energetic significance of their contribution to the network. The correlations in fluctuations between each pair of N-variables of the network are assigned to an N x N diffusion matrix for each microstate. The rate of heat dissipation associated with each composite variable is determined by the magnitude of the fluctuations and the gradient of the landscape. A variable that is a negligible contributor to dissipative heat would be predicted to play a small role in maintaining the network, and could indicate that the variable is coincidental, but not causative, to network function. A possible alternative method to assign relative importance to network components is surprisal analysis, which uses the maximum entropy associated with the time-dependent distribution of variables (51) . To achieve unambiguous interpretation, we

what is the connection here?

show here how these putative relationships can be assessed experimentally by directly measuring trajectories in variable space and their covariance.

This analysis has potential practical implications. $\dot{Q}_{ENERGY}$ is a quantitative measure of the rate of heat produced to maintain a regulatory network. The magnitude of $\dot{Q}_{ENERGY}$ can be used to compare the relative thermodynamic cost of different steady-state phenotypic distributions. For example, as a metric it could provide insight into the thermodynamics of different regulatory networks, or the same network functioning in cells from different individuals. It will be a useful metric to guide cell therapy manufacturing conditions, and to guide the engineering of regulatory pathways in synthetic biology applications.

CONCLUSIONS

Thermo-FP analysis, through rigorous connection to the Boltzmann H function and the rate of dissipation of free energy of the nonequilibrium steady state, provides a direct relationship between composite network variables and their contribution to the heat of maintaining homeostasis of the network.

The application of the Thermo-FP approach allows dynamical analysis of network interactions and cell states on a smooth continuous landscape. A multidimensional (or multi-variable) landscape that considers the dynamics of network components can provide unique understanding of the correct interpretation of cellular phenotypic indicators in the context of other network components, stable attractor states and rates at which neighboring phenotypic states can be accessed. Furthermore, we have shown how $\dot{Q}_{ENERGY}$, the dissipative heat required to maintain a multi-variable network can, in

principle, be determined from experimentally tractable data consisting of a steady state distribution and a diffusion matrix of dynamic covariances in network variables. Each eigenvalue/eigenvector of the N-dimensional rotated matrix represents a unique and independent cluster of cooperative interactions of network components, and each constitutes a degree of freedom of the network.

This approach to analysis of the landscape provides unique insight into the components, paths, and the thermodynamic price associated with maintaining a nonequilibrium stationary multidimensional landscape of a regulated network. While we have focused this analysis on the steady state distribution, this approach will also be useful for tracking how cellular populations transition from one steady state to another in response to environmental changes, by helping to identify and quantify interactions between network components during transitions.

what is the improvement to “simpler correlation based methods?”

REFERENCES

1. Huang S. Reprogramming cell fates: reconciling rarity with robustness. Bioessays. 2009;31(5):546-60.
2. Garcia-Ojalvo J, Martinez Arias A. Towards a statistical mechanics of cell fate decisions. Curr Opin Genet Dev. 2012;22(6):619-26.
3. Sisan DR, Halter M, Hubbard JB, Plant AL. Predicting rates of cell state change caused by stochastic fluctuations using a data-driven landscape model. Proc Natl Acad Sci U S A. 2012;109(47):19262-7.
4. Sasai M, Kawabata Y, Makishi K, Itoh K, Terada TP. Time scales in epigenetic dynamics and phenotypic heterogeneity of embryonic stem cells. PLoS Comput Biol. 2013;9(12):e1003380.
5. Morris R, Sancho-Martinez I, Sharpee TO, Izpisua Belmonte JC. Mathematical approaches to modeling development and reprogramming. Proc Natl Acad Sci U S A. 2014;111(14):5076-82.
6. Kaufmann BB, Yang Q, Mettetal JT, van Oudenaarden A. Heritable stochastic switching revealed by single-cell genealogy. PLoS Biol. 2007;5(9):e239.
7. Halter M, Sisan DR, Chalfoun J, Stottrup BL, Cardone A, Dima AA, et al. Cell cycle dependent TN-C promoter activity determined by live cell imaging. Cytometry A. 2011;79A(3):192-202.
8. Battich N, Stoeger T, Pelkmans L. Control of Transcript Variability in Single Mammalian Cells. Cell. 2015;163(7):1596-610.
9. Singer ZS, Yong J, Tischler J, Hackett JA, Altinok A, Surani MA, et al. Dynamic heterogeneity and DNA methylation in embryonic stem cells. Mol Cell. 2014;55(2):319-31.

10. Filipczyk A, Marr C, Hastreiter S, Feigelman J, Schwarzfischer M, Hoppe PS, et al. Network plasticity of pluripotency transcription factors in embryonic stem cells. Nat Cell Biol. 2015;17(10):1235-46.
11. Patsch K, Chiu CL, Engeln M, Agus DB, Mallick P, Mumenthaler SM, et al. Single cell dynamic phenotyping. Sci Rep. 2016;6:34785.
12. Sigal A, Milo R, Cohen A, Geva-Zatorsky N, Klein Y, Liron Y, et al. Variability and memory of protein levels in human cells. Nature. 2006;444(7119):643-6.
13. Chang HH, Hemberg M, Barahona M, Ingber DE, Huang S. Transcriptome-wide noise controls lineage choice in mammalian progenitor cells. Nature. 2008;453(7194):544-7.
14. Kalmar T, Lim C, Hayward P, Munoz-Descalzo S, Nichols J, Garcia-Ojalvo J, et al. Regulated fluctuations in nanog expression mediate cell fate decisions in embryonic stem cells. PLoS Biol. 2009;7(7):e1000149.
15. Gupta PB, Fillmore CM, Jiang G, Shapira SD, Tao K, Kuperwasser C, et al. Stochastic state transitions give rise to phenotypic equilibrium in populations of cancer cells. Cell. 2011;146(4):633-44.
16. Jafarpour F. Cell Size Regulation Induces Sustained Oscillations in the Population Growth Rate. Physical Review Letters. 2019;122(11):118101.
17. Newman JR, Ghaemmaghami S, Ihmels J, Breslow DK, Noble M, DeRisi JL, et al. Single-cell proteomic analysis of S. cerevisiae reveals the architecture of biological noise. Nature. 2006;441(7095):840-6.
18. Balazsi G, van Oudenaarden A, Collins JJ. Cellular decision making and biological noise: from microbes to mammals. Cell. 2011;144(6):910-25.
19. Prill RJ, Vogel R, Cecchi GA, Altan-Bonnet G, Stolovitzky G. Noise-driven causal inference in biomolecular networks. PLoS One. 2015;10(6):e0125777.
20. Kontogeorgaki S, Sanchez-Garcia RJ, Ewing RM, Zygalakis KC, MacArthur BD. Noise-processing by signaling networks. Sci Rep. 2017;7(1):532.
21. Huang S. The molecular and mathematical basis of Waddington's epigenetic landscape: a framework for post-Darwinian biology? Bioessays. 2012;34(2):149-57.
22. Eldar A, Elowitz MB. Functional roles for noise in genetic circuits. Nature. 2010;467(7312):167-73.
23. Li C, Balazsi G. A landscape view on the interplay between EMT and cancer metastasis. NPJ Syst Biol Appl. 2018;4:34.
24. Dunlop MJ, Cox RS, 3rd, Levine JH, Murray RM, Elowitz MB. Regulatory activity revealed by dynamic correlations in gene expression noise. Nat Genet. 2008;40(12):1493-8.
25. Pakka VH, Prugel-Bennett A, Dasmahapatra S. Correlated fluctuations carry signatures of gene regulatory network dynamics. J Theor Biol. 2010;266(3):343-57.
26. Cox CD, McCollum JM, Allen MS, Dar RD, Simpson ML. Using noise to probe and characterize gene circuits. Proc Natl Acad Sci U S A. 2008;105(31):10809-14.
27. Azhar F, Bialek W. When are correlations strong? arXiv. 2010;1012.5987v1.
28. Rizzino A, Wuebben EL. Sox2/Oct4: A delicately balanced partnership in pluripotent stem cells and embryogenesis. Biochim Biophys Acta. 2016;1859(6):780-91.
29. Weinreb C, Wolock S, Tusi BK, Socolovsky M, Klein AM. Fundamental limits on dynamic inference from single-cell snapshots. Proc Natl Acad Sci U S A. 2018;115(10):E2467-E76.
30. Tkačik G, Bialek W. Diffusion, dimensionality, and noise in transcriptional regulation. Physical Review E. 2009;79(5):051901.
31. Hubbard JB, Lund SP, Halter M. Boltzmann's H-Function and Diffusion Processes. J Phys Chem B. 2013;117(42):12836-43.
32. Risken H. Fokker-Planck Equation. In: Risken H, editor. The Fokker-Planck Equation: Methods of Solution and Applications. Berlin, Heidelberg: Springer Berlin Heidelberg; 1984. p. 63-95.

33. Attard P. Non-equilibrium Thermodynamics and Statistical Mechanics: Foundations and Applications: Oxford Scholarship Online; 2012.
34. Prigogine I. Time, structure, and fluctuations. Science. 1978;201(4358):777-85.
35. Courant RH, D. Methods of Mathematical Physics: WILEY-VCH Verlag GmbH & Co. KGaA; 2004.
36. Bergmann PG, Lebowitz JL. New Approach to Nonequilibrium Processes. Phys Rev. 1955;99(2):578-87.
37. Lavenda BH, Santamato E. The Kinetic Analog of Boltzmanns Principle. J Math Phys. 1981;22(12):2926-33.
38. Oono Y, Paniconi M. Steady state thermodynamics. Prog Theor Phys Supp. 1998(130):29-44.
39. Ge H, Qian H. Physical origins of entropy production, free energy dissipation, and their mathematical representations. Physical Review E. 2010;81(5).
40. Speck T, Seifert U. Integral fluctuation theorem for the housekeeping heat. J Phys a-Math Gen. 2005;38(34):L581-L8.
41. Wang J, Xu L, Wang E. Potential landscape and flux framework of nonequilibrium networks: robustness, dissipation, and coherence of biochemical oscillations. Proc Natl Acad Sci U S A. 2008;105(34):12271-6.
42. Rudin W. Real and complex analysis. 3rd ed. New York: McGraw-Hill; 1987. xiv, 416 p. p.
43. Raj A, van Oudenaarden A. Single-molecule approaches to stochastic gene expression. Annu Rev Biophys. 2009;38:255-70.
44. Austin DW, Allen MS, McCollum JM, Dar RD, Wilgus JR, Sayler GS, et al. Gene network shaping of inherent noise spectra. Nature. 2006;439(7076):608-11.
45. Tomioka R, Kimura H, T JK, Aihara K. Multivariate analysis of noise in genetic regulatory networks. J Theor Biol. 2004;229(4):501-21.
46. Pedraza JM, van Oudenaarden A. Noise propagation in gene networks. Science. 2005;307(5717):1965-9.
47. Wilson JA. Volume of n-dimensional ellipsoid. Sciencia Acta Xaveriana. 2010;1(1):101-6.
48. Bajcsy PC, J.; Simon, M. Web Analysis of Big Image Data: Springer International Publishing; 2018.
49. Bhadriraju K, Halter M, Amelot J, Bajcsy P, Chalfoun J, Vandecreme A, et al. Large-scale time-lapse microscopy of Oct4 expression in human embryonic stem cell colonies. Stem Cell Res. 2016;17(1):122-9.
50. Schiebinger G, Shu J, Tabaka M, Cleary B, Subramanian V, Solomon A, et al. Optimal-Transport Analysis of Single-Cell Gene Expression Identifies Developmental Trajectories in Reprogramming. Cell. 2019;176(6):1517.
51. Remacle F, Kravchenko-Balasha N, Levitzki A, Levine RD. Information-theoretic analysis of phenotype changes in early stages of carcinogenesis. Proc Natl Acad Sci U S A. 2010;107(22):10324-9.

SUPPORTING INFORMATION (SI)

SI1a. Thermo-Fokker Planck (Thermo-FP) dynamics

SI1b. Experimental considerations

SI1c. Convergence of the homeostatic heat to an upper bound: A geometric interpretation.

SI1d. The dissipative heat sustaining homeostasis of a network has a lower bound.

Click here to download Figure FIG 1.tif

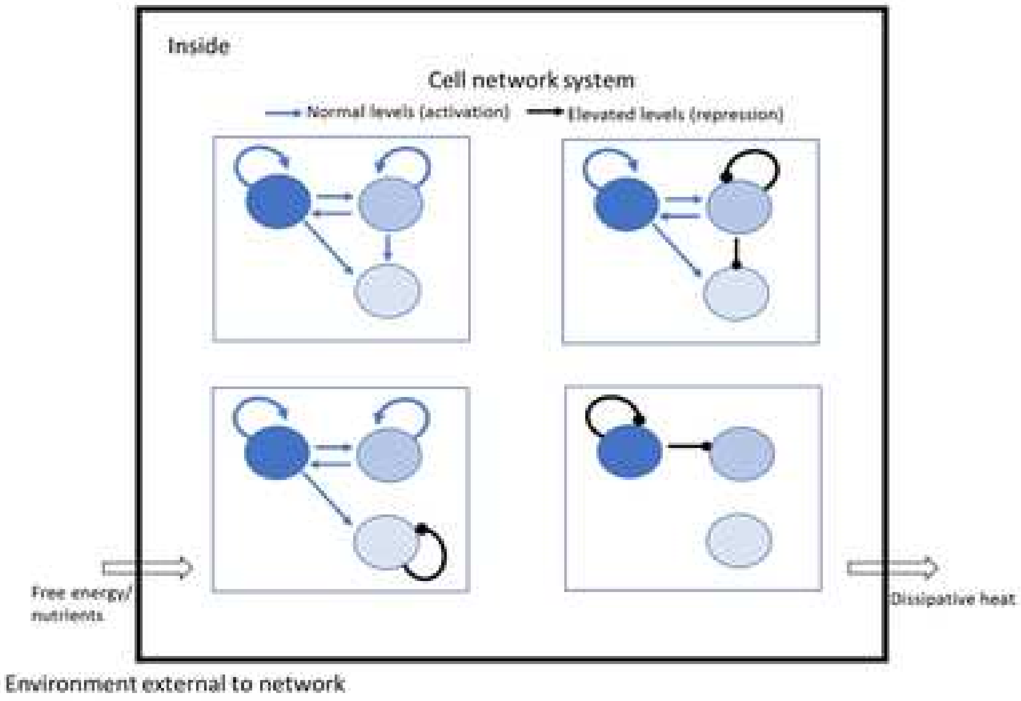

FIGURE 1

figure 2

Click here to download Figure FIG 2.tif

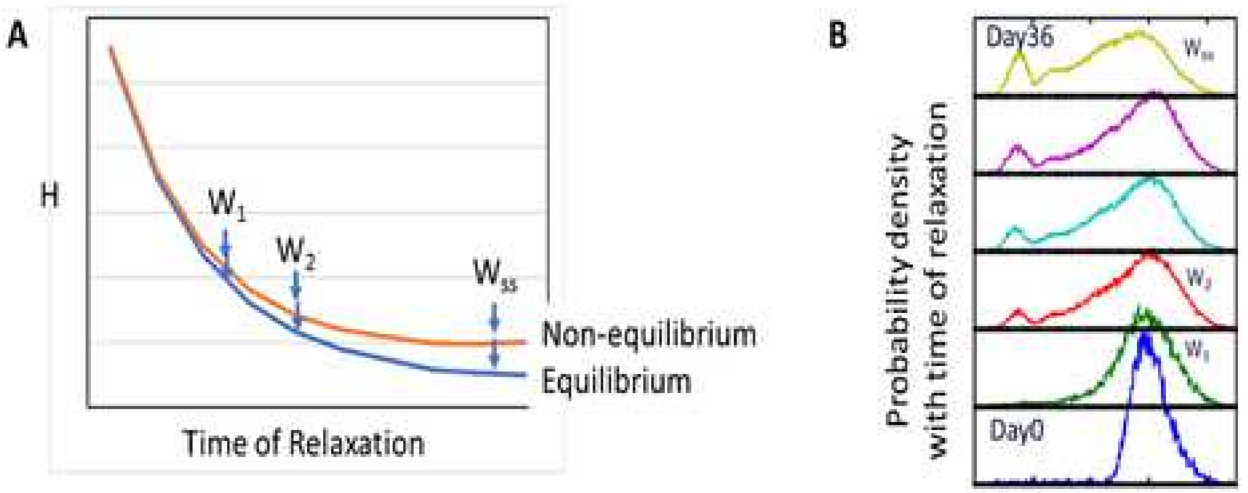

FIGURE 2

Supporting information

Click here to access/download
Supporting Information
SI1_Text.docx
