# Supplementary material for: The role of fluctuations in determining cellular network thermodynamics
Source: PLoS One. 2020 Mar 11;15(3):e0230076. doi: 10.1371/journal.pone.0230076 (PMC7065797; doi:10.1371/journal.pone.0230076)
Supplement: Attachment — Submitted filename: Response2Reviewers.docx [file pone.0230076.s003.docx]

**RESPONSES TO REVIEWERS**

We would like to begin by thanking the Reviewers for their insightful comments, which have helped greatly to improve the manuscript. We have made a number of changes to the manuscript, in addition to the comments and explanations provided below.

In some cases, we have combined related Reviewer comments to provide a complete and concise response. We have also addressed the comments that Reviewer 1 provided in a pdf mark-up of the manuscript; these changes are embedded within the manuscript.

Responses to Reviewer 1

The term Thermo-FP is a bit irritating…What the authors probably mean with the “Thermo” prefix is a thermodynamic interpretation of the fluctuation correlations? This should be clearly stated and explained in the main text.

Yes, the reviewer is correct, that was exactly what we intended. Thank you for the suggestion. We have made this clear by revising the title of the manuscript and adding additional explanation of the term Thermo-FP in the Abstract and Introduction.

The connection to stochastic thermodynamic (sTD) is not done in the main text (besides one ref to Seifert in another context). In this respect, the statement that “For a nonequilibrium steady state system, the occupation of microstates results from irreversible processes ..” (l. 138) is not correct in sTD processes are reversible! This sentence is ambiguous and has been deleted.

o The KL based definition of the relative free energy is rigorously derived by Rao et al. (<https://journals.aps.org/prx/abstract/10.1103/PhysRevX.6.041064>) and should be mentioned and compared here.
Our decomposition of dH/dt, the rate of free energy degradation, is conceptually identical to the decomposition performed in previous studies including Rao et al. We decompose dH/dt into adiabatic and non-adiabatic components, namely the housekeeping heat and entropy production rate due to transient dynamics. Our work builds on the rigorous KL based definition of the relative free energy derived by Rao. The work of Rao et al is now cited in Results.

o The thermodynamic interpretation of fluctuations wrt to cell states and their stability was also studied by e.g. Chen (<https://www.nature.com/articles/srep00342>) or Huang (<https://journals.plos.org/plosbiology/article?id=10.1371/journal.pbio.2000640>).

- Given these published studies, the innovation of the ms is not really clear (and not clearly stated).

We apparently have not articulated the innovative aspects of our theoretical framework. These innovative aspects are:

1) Our derivation for the housekeeping heat results in a quadratic form representation that provides insight into the driving force and flux that are the origin of the heat dissipation of the nonequilibrium steady state (NESS). In the form presented in Eq. 4 and 5 (previously Eq. 5 and 6), the driving force for the NESS has a clear interpretation, namely, the difference between the potential of the NESS and the corresponding equilibrium distribution (or detailed balance). The quadratic form also makes it intuitive how the fluctuations in network components, as reflected by the diffusion matrix, contribute to the housekeeping heat of the NESS.
2) The quadratic form also lends itself to normal mode analysis, which allows identification of independent coordinated network components, the dominant modes of relaxation (degrees of freedom) of the network, that contribute to the housekeeping heat (see section “Normal Mode representation of the diffusion matrix”). As a result, this analysis uniquely applies the housekeeping heat to the interpretation of the organization of cellular networks. This implies that there is a minimum heat associated with correlated fluctuations because the quadratic form is bounded by eigenvalues of the diffusion tensor.
3) Fokker-Planck dynamics makes it straightforward to use experimental data quantifying the phenotype of cells and cell populations to analyze complex network analysis. Data on dynamic covariances in phenotype from quantitative live cell microscopy are becoming increasingly available and the corresponding landscape gradients can be obtained from steady-state cell population data.

We have modified the manuscript (Introduction, Results, Discussion and Conclusions) to more clearly highlight these innovative aspects.

We have also integrated the references brought to our attention by the reviewer in the Discussion section.

The work of Chen et al. and Huang et al. and others are in the same spirit as our theoretical approach in that they apply dynamical systems models to interpret transient behavior of cell populations. While it is not unusual to derive thermodynamic quantities from these types of models, our formulation of the housekeeping heat through experimental measurements of dynamic covariances is novel.  Furthermore our approach is a method to apply the housekeeping heat to interpret the organization of cell networks. With respect to Chen et al. and Huang et al. our work differs in two important ways:

1) The theoretical approaches of Chen et al and Huang et al use the ensemble averages of cell population distributions, while our approach uses the dynamic covariances in phenotype that can be observed in individual cells, as well as the shape of the population distribution. By measuring changes in phenotype (such as expression of a fluorescent protein) in the same living cell over time, one can determine the fluctuation rate for that phenotypic variable and differences in fluctuation rates in different cells. By measuring more than one variable in the same cell, one can measure the correlations in fluctuations for those variables in individual cells, and the differences in correlation in different cells that may be expressing different amounts of one or the other of the variable. Access to dynamic covariance through quantitative live cell microscopy is becoming increasingly available and theoretical frameworks that can be used to interpret cell signaling networks are needed.
2) We show how the shape of the distribution and the dynamic covariances are related to the housekeeping heat required to maintain the NESS (see description of innovative aspects above). Neither Chen et al. nor Huang et al. make connections to this thermodynamic quantity.

- For me the connection from the physical non-equilibrium state to the biological non-equilibrium state is not clearly described. While from the chemo-physical perspective “ENERGY” would correspond to ATP or similar energy substrates, the biological state is here described by the gene regulatory network state. How are these two levels linked? Is the physical heat dissipation comparable with biological ordering? In this context, Eqs. 3-6 should be explained in more detail to enable plausibility check. Why should Eq. 4 be general valid?

Our mathematical framework is applicable to both physical and biological nonequilibrium states. The constraints on the biological system could be considered to be equivalent to boundary conditions.

The dissipative heat is indeed a result of biological ordering and the reduction of entropy. This concept is well developed by Prigogine (citation included in the text). The network is maintained by injection of energy sources or other biochemicals into the system to drive the reactions that maintain the organization of network components, and dissipative heat is released. The advantage of coarse graining with FP is that the molecular species that are involved in maintaining the network variables at their appropriate levels don’t have to be stipulated. In fact, the biological data that are collected (for example by fluorescence microscopy) provide measurable quantification of the response of network variables that could be the result of many upstream (unidentified) factors.

The levels of network variables are measured directly in each individual cell over time; no assumptions of how the microstates are reached are necessary. The coordinated fluctuations between network variables provide the thermodynamic quantities associated with their interdependence as a function of microstate on the landscape. Eq. 4 and 5 (formerly 5 and 6) show how the coordinated fluctuations in network variables (Dij) contribute to the housekeeping heat required for maintaining the nonequilibrium steady state of the network.

In order to connect more seamlessly with other authors, We have removed the term Q.ENERGY and replaced it with Q.HK, the dissipative heat that remains associated with a nonequilibrium steady state after (dH(t))/dt has reached zero. We also modified Eq. 3 so that it is directly comparable to the housekeeping heat described by previous authors (Oono and Paniconi).

- Given these critical comments above, the proposed application to biological systems is interesting but without a proof of concept application to investigate the network contribution (even with public available data) and a concrete comparison with other methods including those mentioned above, the applicability of the suggested framework cannot be judged.

In Sisan et al (PNAS 2012), we showed how experimental data can be used to compute gradients of the landscape and diffusion coefficients, and therefore, in the context of this work, how to compute housekeeping heat. This work follows on that study (which was on a 1-dimensional system) and describes how experimental data from multiple network variables can be collected and interpreted with respect to thermodynamic quantities. In order to make this connection between this work and the previous work, we have elaborated on the methods and results of Sisan et al in the Introduction. In that work, we showed that subpopulations that were each expressing different levels of the reporter relaxed to the steady state landscape with very different kinetics. Langevin/FP dynamics and the diffusion coefficient calculated from experimentally measured fluctuations in fluorescent protein expression allowed nearly perfect prediction of the relaxation kinetics in the absence of any adjustable parameters. This fairly remarkable finding encouraged us to develop the Thermo-FP framework for a multi-variable network system.

Responses to Reviewer 2

This manuscript aims to develop a framework for the thermodynamics of a cell population at nonequilibrium steady states. The analysis referred to as Thermo-Fokker-Planck gives insight into the relative contributions of various network components to the relaxation process. The original method was developed for nonequilibrium steady states of nonliving systems. Efforts are made to apply this method to a population of living cells.

The current theoretical work is based on previous experimental work (Sisan et al, 2012 PNAS) of the cells in a **living** population. Thus, all our efforts are applicable to living systems. In order to make the connection between this work and previous work, we have elaborated on the methods and results of Sisan et al in the Introduction.

(1) The way this method transfers from nonliving to living systems is unclear. What exactly are the sources and sinks of entropy and energy? In what sense and why are cell populations nonequilibrium? What does temperature mean here? Some clarifications are needed to make all this useful for the community.

The Results section on *The steady state landscape and the Boltzmann H function* has been substantially edited to focus better on the thermodynamic aspects of the nonequilibrium steady state that is of primary importance here.

In this work, sources and sinks are the chemical and biochemical conditions that break detailed balance and are the origin of housekeeping heat. In order for cellular networks to maintaining homeostasis, sources of energy or other biochemicals are injected into the system and dissipative heat is released to the exterior of the system as a result. We can speculate about the identity of sources and sinks, but FP coarse graining allows analysis of the thermodynamic contributions of the network without knowing those details. The dynamic data that are collected from living cells over time (for example by fluorescence microscopy) provide a quantification of the response of multiple network variables simultaneously; these responses could be the result of many upstream (unidentified) factors. The advantage of coarse graining with FP is that the molecular species that are involved in maintaining the network variables at their appropriate levels (which are largely unknown) don’t have to be stipulated to gain predictive insight into the parameters of the network.

We have added a citation in the introduction to support the statement: *Living cells are a clear example of a nonequilibrium system* (Wang 2015 Adv Phys). This citation provides many examples of nonequilibrium thermodynamics studies of cells and cell populations.

In addition, we now make clear that by temperature we mean a real thermodynamic temperature; T is now explicitly written into the equations throughout the manuscript. We assume our systems are isothermal and that no entropy production is associated with temperature gradients. We have included this in the paper in Results.

(2) While the manuscript follows the spirit of theoretical papers such as Ref. 38 by Oono & Paniconi, it should also incorporate the spirit of Shin-ichi Sasa & Hal Tasaki, Journal of Statistical Physics 125(1), 2006, which should be cited. What Sasa & Tasaki exemplifies is how very simple, realistic systems such as sheared flow or thermal flow can be used to demonstrate the applicability of theory. The same should be done here for at least one or two biological systems: what plays the role of a “wall” (as in sheared flow) for cell populations

The concentration of biomolecules that break detailed balance (i.e. the energy sources and starting materials) are analogous to the “wall” in a sheared flow system. The constrained concentrations of biochemical species constitute the sources and sinks in our system.

Regarding an example for demonstration, we refer the Reviewer to Sisan et al (PNAS 2012). In that work, we showed how experimental data can be used to compute gradients of the landscape and diffusion coefficients, and therefore, in the context of this work, how to compute housekeeping heat. This work follows on that study (which was on a 1-dimensional system) and describes how experimental data from multiple network variables can be collected and interpreted with respect to thermodynamic quantities. In order to make this connection between this work and the previous work, we have elaborated on the methods and results of Sisan et al in the Introduction.

(3) There are some statements that often fail in biological, cellular systems. For example, ergodicity (line 47) and detailed balance (lines 193-195) completely fail if protein levels affect the growth rate (and thereby the dilution rate). That is, the steady-state moments of time courses from tracking single-cell lineages over time will differ from steady-state moments over cell populations at any given time. This effect is described in PMID:22511863 and PMID:30341217, which would be worth citing and discussing. The statements about ergodicity and detailed balance should include the limitation that these are valid only if growth rates do not depend on protein levels.

We agree with the reviewer that this is an important point regarding the application of some statistical physics concepts (i.e. ergodicity or detailed balance) to cell populations. Therefore, we have added the following paragraph to the Supporting Information 1B :

“Individual cells in a steady-state population are simultaneously transitioning between microstates and dividing. When the division rate depends on the microstate of the cell (e.g. certain phenotypes have different division rates than others), the moments of the steady-state distribution, W(x), will differ from the moments derived by following a number of individual cells over time (Ref PMID:22511863 and PMID:30341217). A microstate-dependent division rate also means that the landscape derived from the observed steady-state distribution, ln (W(x)), includes the effect of microstate-dependent division rates. An approach for compensating the landscape shape to account for microstate dependent division rates for modeling purposes was shown by Sisan et al (ref Sisan, PNAS, 2012). The landscape transformation was possible because the specific form for the dependence of the division rate on the microstate was identified by quantitative live cell microscopy.“

(4) Line 141: “approaching its nonequilibrium steady state, entropy decreases over time” – this statement should be explained and references should be provided as it is unusual for anyone familiar with standard, classical thermodynamics.

Thank you for catching this typographical error. This sentence now reads “approaching its nonequilibrium steady state, entropy increases over time”.

(5) Figure 2: only the high-sorted population is shown over time. In addition, the unsorted population and the low-sorted populations should also be shown at the same time points.

(7) It would be helpful if the method could be illustrated on a very simple, 1- or 2-dimensional system, such as a constitutively synthesized protein with or without self-regulation or something similar, using actual matrices, probability distributions, etc.

Our previously published experimental work (Sisan et al PNAS 2012) which inspired this current theoretical work is now discussed in greater detail in the **Introduction.** The full data set with unsorted and the low-sorted populations is presented in that paper which is referenced. That work provides the best example of this approach. The experimental data was collected with fluorescence microscopy and flow cytometry, and the time-dependent analysis on live cells provided the data from which fluctuation rates were calculated. The paper demonstrates how the data provided both probability distributions and diffusion coefficients. These parameters alone provided a remarkably accurate prediction of the rate at which subpopulations of cells relaxed to the steady state. That study was of a one-variable system, and this theoretical work demonstrates how to build on that with a multidimensional network of associated variables.

(6) Boltzmann’s constant and temperature do not appear in the formulas of the paper. While it is OK to omit them, their meaning should still be clarified. The approach should be developed with k and T present and then they can be dropped once it is clear what happens with their incorporation. In fact, the temperature here is probably related to the fluctuations of molecule concentrations or cell states, meaning that the temperature may not be identical to the typical “absolute temperature” in statistical physics of nonliving systems. This should be clarified.

(8) Related to the previous comment, the heat terms may not be the usual heat measured in nonliving systems. This should be discussed and a practical interpretation for the heat terms should be provided.

Temperature and k_B_ are now explicitly written into the equations throughout the manuscript. We assume that our systems are isothermal with a reservoir and that no entropy production is associated with temperature gradients. We have included this in the paper after Eq. 1**.** The heat referred to is the usual heat measured in nonliving systems. The expectation is that this will be small and difficult to measure explicitly but is identical to the thermodynamic quantity.

(9) The reason for assuming the “upper bound” (line 291) should be clarified. “Temperature associated with heat generation” etc. is unclear because heat and temperature are unclear (see above).

From a practical point of view it is reasonable to assume that there will be a limit to the rate of heat dissipation. While no physical system can withstand an infinite thermal gradient, it is well established that biological systems can be strongly influenced by temperature (Charlebois et al PNAS 2018). This citation has been added in Results.
