# Supplementary material for: The role of fluctuations in determining cellular network thermodynamics
Source: PLoS One. 2020 Mar 11;15(3):e0230076. doi: 10.1371/journal.pone.0230076 (PMC7065797; doi:10.1371/journal.pone.0230076)
Supplement: Attachment — Submitted filename: Response to Reviewer.2.docx [file pone.0230076.s004.docx]

Response to Reviewer.2

January 30, 2020

We have made an additional edit to the manuscript to address the concern of Reviewer 2. We have made a modification to our definition of *T* in Eq 1 and cited this use by Jarzynski in his seminal 1997 work.

“…and $T$ is the thermodynamic temperature of the network system in contact with an isothermal heat reservoir (40). We also assume that no entropy production is associated with temperature gradients. “

We trust that making it clear that the network is in contact with a thermal reservoir at equilibrium is the point the reviewer wanted us to make.

Respectfully,

Anne Plant

National Institute of Standards and Technology
